# Insights into the Metabolic Adaptations of a Carbapenem-Resistant *Klebsiella pneumoniae* Strain on Exposure to Sublethal Concentrations of Ertapenem

**DOI:** 10.3390/ijms26188988

**Published:** 2025-09-15

**Authors:** Daniel Jaén-Luchoro, Francisco Salvà-Serra, Beatriz Piñeiro-Iglesias, Nachiket Marathe, Edward R. B. Moore, Roger Karlsson

**Affiliations:** 1 Department of Clinical Microbiology, Sahlgrenska University Hospital, Region Västra Götaland, 41346 Gothenburg, Sweden; francisco.salva.serra@ri.se (F.S.-S.); beatriz.pineiro.iglesias@vgregion.se (B.P.-I.); erbmoore@ccug.se (E.R.B.M.); 2 Department of Infectious Diseases, Institute for Biomedicine, Sahlgrenska Academy of the University of Gothenburg, 40234 Gothenburg, Sweden; 3 Centre for Antibiotic Resistance Research (CARe), University of Gothenburg, 40530 Gothenburg, Sweden; 4 Culture Collection University of Gothenburg (CCUG), Sahlgrenska Academy of the University of Gothenburg, 41346 Gothenburg, Sweden; 5 RISE Research Institutes of Sweden, 41346 Gothenburg, Sweden; 6 Institute of Marine Research, NO-5817 Bergen, Norway; nachiket.marathe@hi.no; 7 Nanoxis Consulting AB, 40016 Gothenburg, Sweden

**Keywords:** *Klebsiella pneumoniae*, carbapenem resistance, metabolic adaptation, quantitative proteomics, mass spectrometry

## Abstract

*Klebsiella pneumoniae* strains that are resistant to carbapenems are of great concern. Exposure to low concentrations of antibiotics may influence tolerance to antibiotics. Novel antibiotics and treatment options are thus needed, and this need is exacerbated by the rapid and global spread of antibiotic resistance. In this study, we determined the global proteome changes in a *K. pneumoniae* strain (CCUG 70747) carrying carbapenem resistance genes when exposed to low concentrations of ertapenem. Quantitative proteomics was achieved by the tandem mass tag labeling of peptides generated by trypsin proteolysis and mass spectrometry analysis. Bioinformatics analyses were used to observe changes in protein abundance, as well as the gene ontology (GO) terms and pathways associated with the differentially expressed proteins. The number of proteins detected with significant differential abundance were 87 at the highest concentration applied and 61 in the lowest concentration, all compared with the strain cultured without any antibiotics present. Several of these proteins, as well as the GO terms and pathways associated with the proteins, were linked to mechanisms of antibiotic resistance. However, this strain encodes a carbapenemase and other beta-lactamases, and thus, as expected, presented a reasonably modest adaptation in the global proteome upon exposure to the low concentrations of ertapenem applied. Nevertheless, our study identifies pathways that may lead to adaptation under sublethal concentrations of antibiotics leading to strains with higher tolerance.

## 1. Introduction

Carbapenems are last-resort antibiotics widely used to treat extended-spectrum beta-lactamase (ESBL)-producing strains of the family *Enterobacteriaceae*; but the misuse of these drugs has led to the emergence of carbapenem-resistant bacterial strains [1,2,3]. Carbapenem-resistant *Enterobacteriaceae* have been identified by the World Health Organization (WHO) as one group of bacteria of the top-priority pathogens, for which new drugs are urgently needed [4,5,6]. Infections caused by carbapenem-resistant enterobacteria, as well as the mortality rate, have increased significantly in recent years, generating a substantial economic impact on healthcare systems [7,8,9]. Consequently, carbapenem-resistant bacteria represent a serious threat to human health worldwide [1,9]. Among the family *Enterobacteriaceae*, carbapenem-resistant *Klebsiella pneumoniae* (CRKN) represents one of the most prevalent bacterial pathogens around the world, with an estimated mortality ranging from 33 to 42% [10,11,12]. In fact, CRKN is the most prevalent cause of infection of this group of bacteria in China, the United States, and Europe [13,14,15,16]. *K. pneumoniae* is the third-leading pathogen causing deaths associated with antimicrobial resistance [17].

*K. pneumoniae* strains can survive and persist in human reservoirs, as well as environments exposed to water or humidity. The latter environments represent a possible route for the bacteria to spread throughout hospital environments [18,19,20]. This fact, together with *K. pneumoniae* being considered a pathogen with a high capacity of clonal expansion and exchange of mobile genetic elements, increases the risk of outbreaks and the promotion of the spread of antimicrobial resistance genes [3,8,21]. Additionally, *K. pneumoniae* has the capacity of forming biofilms [22,23,24], which increase the natural resistance of bacteria, providing *K. pneumoniae* with enough protection to survive disinfection procedures and make the eradication or treatment of infections challenging [8,24,25].

Overall, CRKN strains have two main mechanisms of carbapenem resistance: carbapenemases, which are the most common and prevalent mechanisms; and a combination of beta-lactamases able to degrade cephalosporins (AmpC, DHA-1, CMY-2, or ESBLs) [26,27]. Moreover, it is crucial to gain deeper knowledge about the mechanisms of pathogenesis and adaptation to antibiotic resistance. This would help us to better understand what happens to the bacterial cell when exposed to antibiotics, beyond the well-known mechanisms. For instance, recent studies have shown that the overexpression of genes such as *bla*_OXA-23_ in *Acinetobacter baumanii* generates antibiotic resistance but also causes cellular damage, especially at the peptidoglycan level [28].

While the dose of antibiotics to treat bacterial infections is high (lethal dose), bacteria can be exposed to nonlethal concentrations, for example, in nature or hospital environments. Antibiotics are frequently detected in water, soil, and sediments due to agricultural activities, pharmaceutical production, and wastewater discharges [29,30,31,32,33,34,35]. Even at low concentrations in these environments, antibiotics exert selective pressure, potentially promoting horizontal gene transfer, disrupting microbial communities, reducing biodiversity, changing virulence patterns, but also altering bacterial morphology and promoting antibiotic resistance selection [36,37,38]. If little is known about how bacteria counteract antibiotics beyond well-defined mechanisms, even less is understood about their responses to low concentrations of antibiotics [38,39,40,41], especially in the short term. Understanding these primary mechanisms, and which proteins or pathways are mobilized, could be key to predicting long-term bacterial adaptation [38], but also in the discovery of weaknesses that could be targets for novel treatment strategies.

The aim of this study was to determine the proteins and pathways that display a significant differential abundance in a multidrug-resistant carbapenemase-producing *K. pneumoniae* strain (CCUG 70747) [42] when exposed to low concentrations of ertapenem in order to understand the background mechanisms involved in supporting antibiotic resistance and adaptation. This study aims to contribute to the broader efforts to understand carbapenem resistance, beyond the well-defined mechanisms already described.

## 2. Results

### 2.1. Sample Overview

The minimum inhibitory concentration (MIC) profile, as well as the specific in-depth MIC determination of ertapenem for the strain *K. pneumoniae* CCUG 70747, was determined using broth microdilution (Tables S1 and S2). Subsequently, two concentrations were selected for downstream analysis: 15.625 µg/mL (1/4 MIC) and 7.8 µg/mL (1/8 MIC). The results obtained at a 1/8 MIC were used as a midpoint reference to monitor how protein abundance evolved as the antibiotic concentration increased.

Samples were analyzed in triplicate for the different conditions (no antibiotic, 1/8, and 1/4 MIC). The strain was exposed to these concentrations of ertapenem at 30 °C for 18 h for a long exposure to sublethal concentrations of ertapenem. After incubation, samples were taken in the stationary phase, processed and analyzed using a proteomic approach. Following the identification and relative quantifications of the expressed proteins, the relative abundances of the identified proteins were analyzed by principal component analysis (PCA) (Figure S1A) and heatmap clustering (Figure S1B) to identify potential outliers among the triplicates for each condition. Sample 2312 (a replicate of 1/8 MIC) was identified as an outlier, in both the PCA and heatmap (Figure S1). Looking into the details of the outlier 2312, abundances demonstrated more extreme values, higher or lower, compared with the other 1/8 MIC samples and samples exposed to no antibiotic (NAb) and 1/4 MIC. This sample was thus removed from further analysis.

A total of 19,424 peptides and 2910 proteins were detected, corresponding to 54.9% of the proteins encoded by strain CCUG 70747. The sample preparation and digestion strategy allowed the analysis of the whole cell proteome. However, membrane proteins may be underrepresented due to their hydrophobic nature. The difference in protein abundance (higher or lower) was calculated by comparing each protein’s abundance under each condition to its abundance without antibiotic (NAb) exposure to calculate the fold change (FC). A significant difference in abundance was considered if the FC was equal to or higher than 1.5 for higher abundance, and equal to or lower than −1.5 for lower abundance. Only results with a *p*-value ≤ 0.05 (Welch’s *t*-test) were considered (File S1). According to this, the maximum number of proteins detected with significant differential abundance were 87 (1/4 MIC vs. NAb) and 61 (1/8 MIC vs. NAb) and overall, more proteins were overexpressed for both concentrations of antibiotics when compared with NAb (Table 1). When 1/4 MIC is compared with 1/8 MIC, only six proteins had significantly higher abundance (File S1).

**Figure 1:**
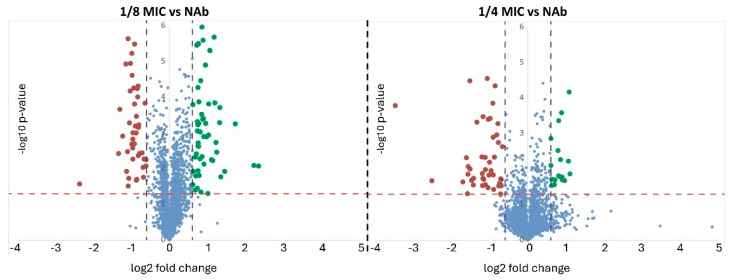
Volcano plots showing the −log10 (*p*-values) vs. log2 (fold change). The horizontal red line indicates the threshold, above which the proteins are demonstrating significant *p*-values, whereas the two vertical blue lines indicate the thresholds for the fold changes ±1.5. In red: proteins with significant lower abundance; in green: proteins with higher abundance; in blue: proteins below thresholds of FC and/or *p*-value.

**Figure 2:**
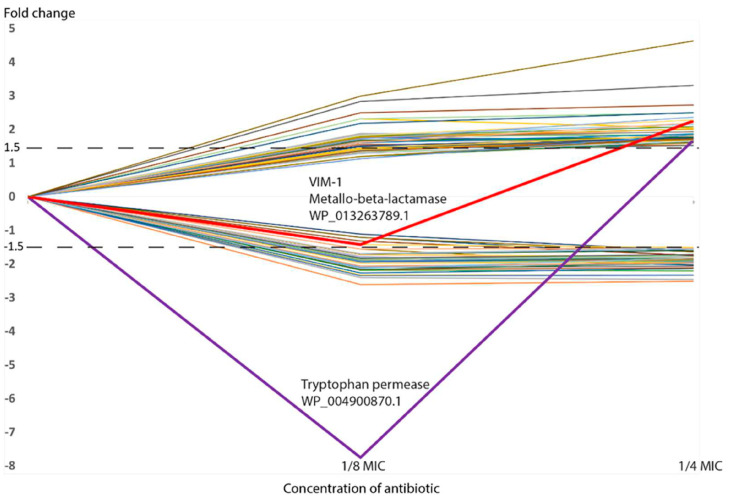
Trends of differential protein abundance. Each line represents a specific protein and only those proteins that were differentially expressed at 1/4 MIC are shown. Dotted lines represented FC ± 1.5.

Volcano plots were generated, showing the evolution of the differential abundance going from 1/8 MIC to 1/4 MIC (Figure 1). In general, there was an increase in the number of proteins passing the set thresholds, the overall FCs, and the statistical significance when going from 1/8 MIC to 1/4 MIC. Regarding proteins demonstrating significant differential abundance (passing threshold of ±1.5 FC and *p*-value ≤ 0.05), most proteins were showing a consistent trend when observing both sub-MICs versus NAb, displaying either an increasing or decreasing trend in their abundance as the concentration of antibiotic increases (Table 2 and Table 3 and Figure 2). However, two proteins do not follow this pattern and show a lower abundance at 1/8 MIC and then a higher abundance at 1/4 MIC (Table 2, Figure 2): one is VIM-1 (WP_013263789.1, a subclass B1 metallo-beta-lactamase); and the other is a tryptophan permease (WP_004900870.1), which has also been proposed to play a role in antibiotic resistance [43]. The trends of all differentially expressed proteins were represented in Figure 2, clearly showing the different trends of these two proteins with respect to the others.

### 2.2. Functional Categorization of Significantly Different Expressed Proteins

**Figure 3:**
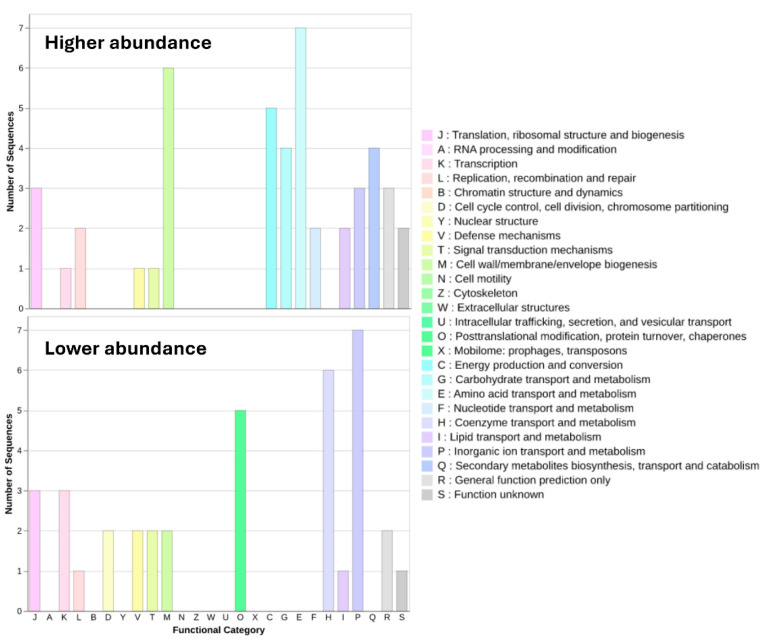
Functional categorization of proteins with significant changes in abundance.

The proteins displaying significant differential abundance levels at the highest concentration used (Table 2 and Table 3) were functionally classified, using the cluster of orthologous groups (COG) database [44] (Table S3, Figure 3), to determine which functional categories were the most affected in each case. The two most represented categories were “Inorganic ion transport and metabolism” (P) and “Function unknown” (S), with 11 and 10 differentially expressed proteins, respectively (Figure 3).

**Figure 4:**
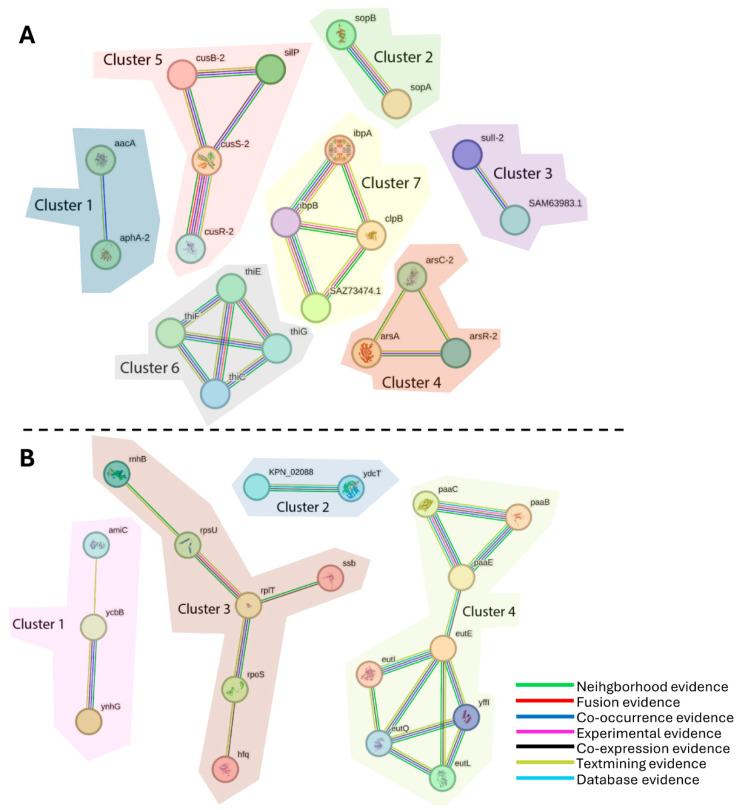
STRING network showing interactions of proteins that demonstrate (**A**) lower abundance and (**B**) higher abundance at 1/4 MIC compared to no antibiotic exposure.

STRING analyses were also performed for the differentially expressed proteins at 1/4 MIC. There were seven clusters found related to proteins with lower abundance (Figure 4A and File S2) and four clusters related to proteins with higher abundance (Figure 4B). In the first case, cluster 1 and cluster 3 were directly linked with antimicrobial resistance (AMR): SAM63983.1 (cluster 3) which is involved in the response to antibiotics (streptomycin, spectinomycin) and refers to the protein encoded by *ant(3″)-Ia* (UniProt accession number Q7B8A0); while AphA-2 (cluster 1) is related to the response to aminoglycosides [45]. Moreover, the thiamine cluster (cluster 6) is related to virulence [46] and in cluster 7, IbpA, and IbpB are related to stress response [47].

On the other hand, regarding clusters of proteins with higher abundance (Figure 4B and File S2), two of the clusters had members linked to AMR (cluster 1 and cluster 4). In cluster 4, we observed the Phenylacetic acid (PAA) sub-cluster (PaaB, PaaC, PaaE), the pathway of which has been shown to play a role in pathogenicity and antibiotic resistance [48], whereas in cluster 1, the protein YcdB [49], a putative amidase, which is described as a Glyoxalase/Bleomycin resistance protein/Dihydroxybiphenyl dioxygenase and a metallo-beta-lactamase, and AmiC and YnhG are linked to resistance against cationic antimicrobial peptides (CAMPs) [50]. Finally, RpoS was found in cluster 3, a global sigma factor related with stress response during stationary phase and biofilm formation [51].

### 2.3. GO Terms and Pathway Enrichment Analysis

**Figure 5:**
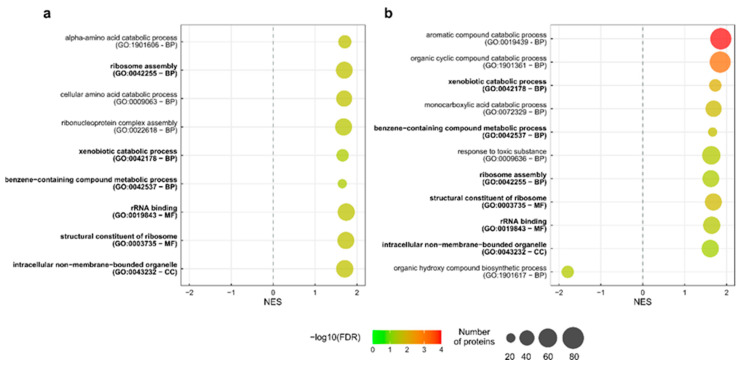
Bubble plot representation of the GO terms that were enriched in *K. pneumoniae* CCUG 70747 in (**a**) 1/8 MIC and (**b**) 1/4 MIC conditions. The color of the bubbles signifies statistical significance, whereas the size of the bubbles signifies the number of proteins having a specific GO term. In bold: GO terms enriched in both conditions. BP, Biological Process; MF, Molecular Function; CC, Cellular Component; FDR, false discovery rate; NES, normalized enrichment score.

The strain CCUG 70747 highlighted several GO terms associated with Cellular Compartment (CC), Biological Process (BP), and Metabolic Functions (MFs) enriched with proteins with higher abundance in both 1/8 MIC and 1/4 MICs, while only one BP-related GO term was identified enriched with a lower abundance in 1/4 MIC: GO:1901617 (organic hydroxy compound biosynthetic process) (Figure 5, File S3).

Regarding the enriched metabolic pathways, CCUG 70747 showed a metabolic pathway related to amino acid metabolism (KO00360 Phenylalanine metabolism) and genetic information processing (KO03010 Ribosome) with higher abundance in 1/8 MIC. These two pathways were also associated with higher abundance in 1/4 MIC together with biofilm formation (KO05111) (File S3). On the other hand, only the pathway KO00730 (thiamine metabolism), related to metabolism of cofactors and vitamins, was found to have lower abundance in 1/4 MIC, which was also found in the STRING analysis (Figure 4A, cluster 6).

## 3. Discussion

Carbapenem-resistant bacteria are currently a great concern worldwide due to the challenges faced in the treatment of infections [2,3,4,5,6,7,8,9,10,52]. This is especially important in CRKP, as *K. pneumoniae* represents a major menace among carbapenem-resistant enterobacteria [8,12]. Even though carbapenemases or the modification of cell wall permeability are well known carbapenem resistance mechanisms [53], little is known about other molecular mechanisms or pathways that play a role in the activation of resistance mechanisms in the presence of carbapenems [54], especially when exposed to low concentrations of antibiotics. This study was designed to provide more insights into the systemic responses to low concentrations of ertapenem, with the objective of understanding alternative mechanisms that may influence AMR and may be used in the future development of alternative treatments. For this purpose, this study has focused not only on proteins with significant abundance variation, but also on analyzing the entire detected proteome to identify possible enriched mechanisms or functions that, even with subtle changes in protein levels, could potentially impact antibiotic resistance. *K. pneumoniae* strain CCUG 70747 was part of a previous study by Johnning et al., 2018 [42]. The purpose of the present study was to perform in-depth proteomic profiling as a response to exposure to antibiotics to further understand the remodeling of the proteome. Admittedly, a limitation of the study is the inclusion of a single strain only, and as CRKP strains are a diverse group [55], additional work is required to capture any shared trends in proteomic changes, including the analysis of many representative strains.

When exposed to 1/4 of its MIC of ertapenem, strain CCUG 70747 showed a total of 87 proteins with significant differences in abundance functionally related to the cell wall, protein production and turnover, amino acid metabolism, energy production, and other processes. The differential abundance of its main carbapenem resistance mechanism, VIM-1, was also observed, along with other proteins involved in resistance to related CAMPs. Even though the mobilization of strategic proteins could lead to resistance, it is worth highlighting that the higher production of carbapenem resistance proteins has been associated with a significant impact on fitness costs [56]. Whether the proteins identified in the present study have a specific role in resistance or impact on fitness cost would need further investigation. Regarding the membrane and cell wall, only mild adjustments were seen in influx–efflux capacity, but key proteins were mobilized to maintain cell wall integrity. Biofilm formation did not show significant changes in protein abundance; however, it was detected as an enriched KEGG pathway. Overall, the cell exhibited a mild proteomic adjustment, with notable changes in key functional areas that may contribute to survival in the presence of ertapenem.

### 3.1. Functional Categories

The functional classification of the proteins with significant differences in abundance of the strain showed that there were opposing trends in the most represented functional categories (Figure 3, Table S3). For instance, most of the differentially abundant proteins within the COG categories “Coenzyme transport and metabolism” (H), “Posttranslational modification, protein turnover, chaperones” (O), and “Inorganic ion transport and metabolism” (P) had a lower abundance compared with no antibiotic exposure, whereas most of the proteins within the categories “Cell wall/membrane/envelope biogenesis” (M), “Energy production and conversion” (C), and “Amino acid transport and metabolism” (E) showed higher abundance (Figure 3). The category “Translation, ribosomal structure and biogenesis” (J) had the same number of proteins with higher and lower abundance. Previous studies have shown effects on the functionalities reflected in categories O and C, observing a differential abundance of proteins involved in energy metabolism, as well as stress proteins involved in ensuring the correct folding or re-folding of proteins. It was suggested that this may respond to the higher energy demands as well as ensuring the correct protein folding during stress conditions [54]. Interestingly, the five proteins classified in category O in the present study, which correspond to five heat shock proteins and one Clp protease, showed lower abundance (Table S3). Category E may respond to ensure a sufficient supply of amino acids to the higher protein production demand. Category M may respond directly to the presence of ertapenem and its detrimental effect on the cell wall. In the presence of beta-lactam antibiotics, the cell tries to compensate for damage caused and ensure the cell wall integrity, which is an effect that has been observed, among others, in the presence of meropenem and cefadroxil [54,57]. It is worth highlighting that a few proteins were classified in the category “Function unknown” (S). “Hypothetical proteins”, or any protein for which the function is not yet described, represent a great source of new functions that could be related, if they show changes in abundance, to the responses of the cells to antibiotics. Interestingly, similar observations have been made in other bacterial species, where proteins of unknown function may influence adaptation to stress and antibiotic exposure [57,58]. These findings may suggest that these hypothetical proteins could help in survival under selective pressures and could represent yet unexplored mechanisms of resistance or tolerance. Unravelling the specific role of these proteins, as well as confirming if changes in abundance are due to the presence of the antibiotic, could represent a great step forward in the better understanding of new factors to consider in the antibiotic resistance phenotype. A good example of this potential was the identification of an uncharacterized protein belonging to the LysM domain/BON superfamily protein that was later suspected to be related to the stress response towards carbapenem [54,59]. Nevertheless, further work will be necessary to investigate the function of the proteins of unknown function found in the present study, and if that function is related or not to antibiotic resistance.

### 3.2. Enriched Metabolic Pathways and GO Terms

The analysis of metabolic pathways through Gene Set Enrichment Analysis (GSEA) was performed on the annotated pathways with the entire set of detected proteins, and not only the proteins that passed the established thresholds. Instead, a weighted value was used, considering both the FC and associated *p*-value of each protein. This approach aimed to assess whether specific pathways showed a common trend in all proteins with variation in their abundance, even if they were only slightly altered.

Regarding GO terms related to BP, in the 1/8 MIC, “Alpha-amino acid catabolic process”, “Ribosome assembly”, “Cellular amino acid catabolic process”, “Ribonucleoprotein complex assembly”, “Xenobiotic catabolic process”, and “Benzene-containing compound metabolic process” were enriched with proteins with higher abundance. At this concentration, the bacterial cell seems to be focusing on amino acid catabolism and ribosome formation, which could be related to a higher demand of protein turnover. The metabolism of alpha-amino acids, such as arginine or its derivative proline, have been identified as differentially expressed in other studies as a consequence of antibiotic stress, and have been related to biofilm formation [60], structures that tend to increase bacterial tolerance to external agents. The BP GO terms enriched in the 1/4 MIC are “Aromatic compound”, “Organic cyclic compound”, “Xenobiotic and monocarboxylic acid catabolic processes”, as well as “Organic hydroxy compound biosynthetic process”, which shows lower abundance. The two most represented BP are “Aromatic compounds” and “Organic cyclic compound catabolic processes”, which are enriched only at 1/4 MIC. This may point to the high importance of these processes in strain CCUG 70747 with the highest concentration of antibiotic used. Interestingly, the GO term “Response to toxic substance” was also represented with a considerable number of proteins (File S3). This could be related directly to the presence of antibiotics or to reducing the toxic compounds generated due to the stressful situation.

If 1/8 MIC and 1/4 MIC conditions are compared, they present three BPs in common, which are “Ribosome assembly”, “Xenobiotic catabolic process”, and “Benzene-containing compound metabolic process”. Considering xenobiotics as substances that are not naturally present in a specific organism, [61], this could be directly related to the presence of the antibiotic entering the cell, or to the increase in the concentration of compounds derived from the action of the antibiotic or the answer of the cell to its presence. In other words, this could be related to the cell attacking the antibiotic or trying to control the concentrations of secondary compounds related directly or indirectly to the presence of the antimicrobial compound. Regarding GO terms related to MFs and CC, both conditions showed the same enriched GO terms: two Ribosome-related MFs and “Intracellular no-membrane-bounded organelle” as a CC GO term, with apparently similar numbers of proteins in all cases. Overall, it is evident that the GO terms detected in the 1/4 MIC condition indicate a higher level of stress, probably due to the higher antibiotic concentration. Finally, the GO term associated with the BP “Organic hydroxy compound biosynthetic process” was found to be less expressed.

Another strategy that bacteria use to protect themselves from environmental stress is the generation of biofilms [22,23,24,53,62]. Biofilms provide an environment where bacterial cells show more resistance to antibiotics than in a free-living form [22,62]. A previous proteomic study has identified biofilm-related proteins whose abundance varies in the presence of carbapenem, suggesting a possible link to the cell’s attempt to increase its tolerance to the antibiotic [63]. In the same study, the CRKP strain was exposed to ertapenem for shorter periods of time, which may allow for the identification of differentially expressed proteins associated with an early response. The strain CCUG 70747 did not present any significant variation in the abundance of proteins linked to biofilms in terms of proteins with a higher or lower abundance, but it was exposed to stress conditions longer. However, a “biofilm” pathway (KO05111) was identified as enriched at 1/4 MIC in the pathway enrichment analysis, even though individual proteins did not pass the thresholds established in the present study. The low level of stress may slightly mobilize this pathway after long-term exposure, potentially making the strain more prone or preconditioned for biofilm formation if the concentration increases.

Taken all together, the changes reflected in the few enriched pathways identified suggest an overall mild adaptation to the presence of ertapenem. Other studies exposing *K. pneumoniae* to similar concentrations of ertapenem show significantly higher changes in their overall metabolism, both in the pathways and number of proteins with significant higher or lower abundance [63]. Other examples, such as a study exposing *K. pneumoniae* to imipenem, meropenem, and colistin, or a study on the adaptation of *P. aeruginosa* to meropenem, also show a much stronger impact on metabolic adaptation. These studies report hundreds of proteins displaying significant differential abundances and a broader range of enriched metabolic pathways, even at the lowest concentrations tested [64,65].

### 3.3. Beta-Lactamases and Other Antibiotic Resistance Genes

Regarding the abundance of β-lactamases and other antibiotic resistance genes, only the carbapenemase VIM-1 showed a significantly higher abundance (FC 2.25), while the carbapenemase KPC-2 and the beta-lactamase SHV-11 were slightly more abundant when compared with the absence of ertapenem, but under the thresholds of significance. SHV-200 was not detected in these conditions. VIM-1 and KPC-2 are both efficient carbapenemases and represent the major resistance mechanism of the strain. In this case, the hypothesis could be that a slight increase in abundance in these major genes, when increasing the concentration of the antibiotic, could represent a strong frontline to counteract the effect of ertapenem. Hence, there might be little need for further adjustments of the global proteome, at least under the concentrations applied in this study. Regarding other resistance genes of strain CCUG 70747, products of resistance genes *sul1*, *acc(6′)-Ic*, *dfrA1*, and *ant(3′′)-Ia* demonstrated a significantly lower abundance compared with NAb (Table 3). The latter three genes are encoded in the same cassette as VIM-1, while *sul1* is encoded around 55 kb away in the same plasmid. This trend could be seen at the protein level in the STRING cluster network (Figure 4), with SAM63983.1, a protein encoded from *ant(3′′)-Ia* and protein AphA-2 showing lower abundance (Figure 4, File S2) when compared with NAb to ertapenem. On the other hand, the proteins with higher abundances and linked to AMR seen in the cluster STRING (Figure 4, File S2) were PaaC, PaaB, and PaaE (cluster 4); the Phenylacetic acid (PAA) pathway linked to AMR [48]; the YcbB (cluster 1), a putative amidase linked with beta-lactamase resistance [49]; and proteins AmiC and YnhG, which are linked to CAMP resistance (cluster 1) [50]. The differential abundance of proteins related to the CAMP pathway under antibiotic stress have been described previously [66] and proposed as interesting targets for tackling CRKP infections [64].

### 3.4. Adjustment of the Influx–Efflux Capacity

Concerning the transport capacity through the cell wall, CCUG 70747 mobilizes at least nine proteins under the conditions studied (1/4 and 1/8 MIC). Porins seem to be one of the main routes that beta-lactams use to enter the cell [63]. OmpK35 and OmpK36 are the two major porins in *K. pneumoniae* [67,68,69], in which mutations have been related to antibiotic resistance [68,69,70,71,72,73]. In the case of OmpK35, mutations tend to truncate the gene, generating a non-functional protein, while in the case of OmpK36, either its abundance is reduced or the pore diameter is constrained [71,73,74]. A proteomic study performed by Yuan et al. [63] exposed a CRKP strain, whose carbapenem resistance is linked with the mutations in OmpK35 and OmpK36, to ertapenem. The study observed an increased abundance of up to nine types of ABC transporters associated with the transport of different types of molecules, as well as several porins. The same study suggested that these adjustments were a response to the loss of the major porins and the change in the consumption of carbon sources [63]. These major porins are encoded in strain CCUG 70747, and a previous study did not detect any mutations in Loop3 of OmpK36 and did not identify a truncated OmpK35 [74]. That means that the strain most probably encodes for wild-type versions of the genes, which is confirmed when the sequences are compared with the ones present in the reference strain *K. pneumoniae* ATCC 700721 (MGH 78578) (accession number NC_009648.1). Regarding the abundance of OmpK35 and OmpK36 in CCUG 70747, OmpK35 was not detected and OmpK36 has a slightly higher abundance level compared with NAb, although under the thresholds (FC 1.28). Finally, in this strain, OmpN is also encoded but was not detected, and OmpW shows almost no variation in its FC when compared with the absence of antibiotics. The overexpression of *ompN* has been linked to increasing antibiotic sensitivity [75,76], and its downregulation suggested as a factor contributing to antibiotic resistance [75,76], while a lower abundance of OmpW has also been observed in other carbapenem resistant strains [75]. These previous observations may explain why OmpN is undetectable in CCUG 70747 or why there is no variation in OmpW abundance. Additionally, the presence of wild type *ompK35* and *ompK36* genes may contribute to why the strain does not need to balance the abundance of other porins in the same way that CRKP strains deficient in these major porins might need to [67,68], but the cell keeps them at a low level to avoid detrimental effects caused by the antibiotic.

### 3.5. Penicillin-Binding Proteins, Cell Wall, and Peptidoglycan Metabolism

The cell wall surrounding bacterial cells is a key element for protecting bacterial survival. This structure is formed by a sophisticated network of N-acetylmuramic acid and N-acetylglucosamine sugars interconnected by stem peptides, forming the so-called peptidoglycan [77,78,79]. In Gram-negative bacteria, this structure is surrounded by the outer membrane protein and the inner membrane protein [69,78,79]. Obviously, such an important structure needs to be correctly synthesized and maintained, as well as being properly adjusted in response to variations in the environment [77]. Alterations of the peptidoglycan biosynthesis process can lead to fatal consequences for the bacterial cell and, in fact, peptidoglycan biosynthesis is targeted by many clinically relevant antibiotics, such as penicillins [77]. Consequently, the protection of the cell wall is a key factor in order to ensure bacterial survival when being targeted by antibiotics [80], and proteome adjustments regarding protective proteins have been previously reported [64,81]. The strain CCUG 70747 is indeed showing a higher abundance of proteins destined to maintain cell wall integrity, including so-called L-D-transpeptidases, here in this strain identified as YnhG (WP_004143241.1) and YcfS (WP_002900790.1) by BLAST (https://www.uniprot.org/blast, accessed on 1 August 2025) at Uniprot. YnhG (WP_0023280306.1), as well as ErfK (WP_0023280306.1) and YcbB (WP_002898195.1), were all demonstrating an increased abundance during antibiotic exposure (Table 2). YnhG and YcbB are involved in the direct cross-linking of meso-diaminopimelate, i.e., DAP-DAP or 3→3 crosslinks. The most common crosslinks in the peptidoglycan are the 4→3 bonds, established by D,D-Transpeptidases [82]. D,D-Transpeptidases are inhibited by antibiotics of the β-lactam family (penams and cephems), thus inducing impairment on the integrity of the cell wall [77], but this inhibition is not efficient in L,D-transpeptidases. As a defense mechanism in the presence of β-lactams, L,D-transpeptidases genes can bypass the antibiotic effects by being overexpressed, thus increasing the presence of 3→3 crosslinks in the cell wall, ensuring the integrity of the peptidoglycan [78,79,82]. YcfS and YcbB are involved in the covalent attachment of Braun’s lipoprotein, or Lpp, to the peptidoglycan [82,83,84]. The Lpp establishes a link between the peptidoglycan and the outer membrane, thus providing structural integrity to the cell wall [85]. In the presence of ertapenem, strain CCUG 70747 increases the abundance of YcbB, probably contributing to ensuring and stabilizing the integrity of the cell wall. A higher abundance of membrane-related lipoproteins has also been described in other species, such as *Acinetobacter baumannii* under antibiotic stress, and linked to its importance in the completion of membrane biogenesis [58,86].

Regarding other peptidoglycan-related proteins present in strain CCUG 70747, only one amidase (WP_004174538.1) was found to be significantly more abundant, even though some other proteins involved in the process were detected with no significant changes in their abundance. This may suggest that, because of the genetic background, strain CCUG 70747 does not need to adjust the proteome regarding that aspect, probably due to the presence of the set of carbapenemases in this strain.

### 3.6. Regulome

A total of 282 regulatory proteins were annotated in the strain CCUG 70747, including transcription factors (TFs), two component systems (TCSs), and other DNA-binding proteins (OBPs). From these proteins, only 10 were identified with significant variation in their abundance, according to the thresholds established (Table S4). This indicates that just a small proportion of regulatory proteins codified in its genome and identified by P2RP needed a significant variation in their abundance, higher or lower. In strain CCUG 70747, eight out of ten proteins were identified as TFs and two as TCSs (Table S4). Two of the TFs showed a higher abundance upon exposure to antibiotics, while the remaining eight proteins (six TFs and two TCsS) demonstrated a lower abundance. It is worth highlighting that the RamA protein, whose regulon is important in antibiotic resistance [87,88], is codified in the strain, but was not detected at any concentrations tested in this study.

Sigma factors are essential regulatory proteins for transcription initiation. Different sigma factors will regulate the transcription of different subsets of genes by specific promoter recognition and the specific environmental signals being detected by the bacteria [89]. Strain CCUG 74707 shows a higher abundance of the sigma factor RpoS (WP_002915106.1), which is crucial for bacterial survival under extreme conditions, and is involved in the regulation of the expression of genes involved in a wide range of functions, such as stress response and virulence [51,90,91]. Interestingly, an additional sigma factor demonstrates a lower abundance upon exposure to ertapenem. This sigma factor is annotated in GenBank as RNA polymerase sigma factor FecI (WP_004118235.1), which belongs to a subgroup of the sigma 70 family named as the extracytoplasmic function sigma factors [92].

## 4. Materials and Methods

### 4.1. Strain

*K. pneumoniae* CCUG 70747 is a multidrug-resistant clinical strain isolated from a human wound in Gothenburg (Sweden) that was showing low susceptibility to carbapenems. This strain has been previously sequenced and genomically characterized [42] and encodes different antibiotic-resistant genes, including carbapenem-resistant genes (*bla*_VIM-1_, *bla*_KPC-2_, and *bla*_SHV-200_) (Table 4). The strain was provided by the Culture Collection University of Gothenburg (CCUG, Gothenburg, Sweden) and was cultivated aerobically on blood agar medium (Columbia agar base plus 5% defibrinated horse blood) at 30 °C for 24 h. Minimal inhibitory concentrations (MICs) were determined at the National Reference Laboratory for Antibiotic Resistance (Växjö, Sweden) using the broth dilution method, following the EUCAST (European Committee on Antimicrobial Susceptibility Testing) recommendations [93] and the ISO standard 20776-1 (2019) [94]. The MICs of 24 antimicrobial agents included in the “Enterobacterales standard panel” (analysis number 25630) were determined. Additionally, strain CCUG 70747 was tested in-house for higher concentrations of ertapenem, following the same recommendations. Clinical breakpoints were set, according to the EUCAST breakpoint tables v13.0 (2025) [95].

### 4.2. Quantitative Proteomic Analysis

Sample preparation and proteomic analysis was performed as previously described [57] and detailed in File S4. Briefly, fresh biomass was used to prepare a 0.5 McFarland cell suspension to prepare the bacterial working solution. Three ertapenem concentrations were tested: no antibiotic, medium subminimum inhibitory concentration (1/8 MIC), and subminimal inhibitory concentration (1/4 MIC). Minimum inhibitory concentrations were performed to decide the 1/8 MIC and 1/4 MICs. For each condition, three technical replicates were prepared by inoculating three tubes with 4 mL of Mueller–Hinton broth, which were cultivated aerobically at 30 °C for 18 h, with orbital shaking (200 rpm). Samples were subsequently precipitated and washed with phosphate saline buffer (PBS). The final pellet was treated with sodium dodecyl sulfate (SDS) solution 20% and lysed by bead-beating (TissueLyser II, Qiagen, Hilden, Germany). After protein quantification, 30 micrograms of protein was digested with trypsin, and the resulting peptides were labeled using a TMT 10-plex isobaric mass tagging reagent (Thermo Fisher Scientific, Waltham, MA, USA). Samples were fractionated and analyzed using an Orbitrap Fusion™ Lumos™ Tribrid™ mass spectrometer (MS) interfaced with an Easy-nLC1200 liquid chromatography system (Thermo Fisher Scientific, Waltham, MA, USA). Finally, protein identification and quantification were performed using Proteome Discoverer version 2.4 (Thermo Fisher Scientific, Waltham, MA, USA) and the protein sequences extracted from the strain’s genome sequence.

### 4.3. Proteomic Data Analysis

Proteomic data exported from Protein Discoverer was analyzed with R v4.0.3 [96]. Specifically, principal component analysis (PCA) was performed using the prcomp function of the stats package, and plots were generated using ggplot2 [97]. The ComplexHeatmap [98] function was used for clustering samples and generating the respective heatmaps. Fold changes in each protein were calculated by dividing the mean of the relative abundance of 1/8 MIC or 1/4 MICs with respect to the mean relative abundance of the same protein in the no antibiotic conditions. The fold change cut-off was set to ±1.5, signifying a biological response (FC ≥ ±1.5)**.** Welch’s *t*-test *p*-values were calculated from log2-transformed abundances at 1/8 MIC or 1/4 MICs with respect to no antibiotic, using the TTEST function in excel (two-tail distribution and two-sample unequal variance). Volcano plots were generated by plotting the fold changes in each protein against the respective −log10 *p*-value. *p*-values ≤ 0.05 were considered statistically significant. From these cut-off values, it was possible to decide which proteins were significantly and differentially expressed.

### 4.4. Enrichment Analyses and Protein Characterization

Initially, protein sequences were classified into COG categories with COGclassifier v2.0.0 (https://github.com/moshi4/COGclassifier, accessed on 3 May 2025) using the default parameters. A more in-depth analysis was performed using OmicsBox v2.0.36 (BioBam Bioinformatics S.L., Valencia, Spain), as previously described by Salvà-Serra et al., 2023 [65]. Briefly, proteins were classified into GO terms [99,100] by eggNOG-Mapper v2.1.9 [101], IterProScan v5.54-87-0 [102], and Blast2GO [103]. Only terms based on one-to-one ontology and experimental evidence were considered in eggNOG-Mapper annotation. Annotation results were filtered to remove redundancy using the GO True Path Rule and the Class Gammaproteobacteria (Taxonomy ID: 1236). The filtered results were assigned to Enzyme Commission (EC) numbers, and metabolic pathways were annotated with eggNOG v5.0.2 using these EC numbers and the Kyoto Encyclopedia of Genes and Genomes (KEGG) [104] in OmicsBox v2.0.36. To study which GO terms and pathways were enriched after antibiotic treatment with ertapenem, the entire set of proteins detected were analyzed, without applying the FC or *p*-value thresholds.

The GO terms and KEGG pathways obtained previously were used for performing Gene Set Enrichment Analyses (GSEAs) using OmicsBox v2.0.36, as previously described [65]. The analysis enrichment statistic (*p*) was set to 1 and 100 permutations were performed. Default parameters were used for maximum and minimum gene set sizes (500 and 15). Only results with a false discovery rate (FDR) q-value below 0.05 were considered for further analysis. The ggplot2 package was used to plot the results using RStudio v2021.09.2 [105], following a previously described protocol by Bonnot et al., 2019 [106].

## 5. Conclusions

The quantitative proteomic characterization of the carbapenem-resistant strain *Klebsiella pneumoniae* CCUG 70747 exposed to subinhibitory concentrations of ertapenem revealed a moderate but coordinated cellular response. The most notable changes involved proteins related to the cell envelope, amino acid metabolism, and energy production, as well as biofilm capacity, suggesting an adaptive response to β-lactam stress. The relatively limited number of differentially expressed proteins may reflect the strain’s genetic determinants for carbapenem resistance, reducing the need for large-scale proteomic adaptation. Nonetheless, the identified shifts reveal potential alternative mechanisms that support cell survival under antibiotic exposure, potentially making the cell more prepared to respond rapidly and efficiently to an increase in antibiotic concentration. These findings underscore the value of quantitative proteomics in uncovering subtle phenotypic responses in multidrug-resistant bacteria and highlight the importance of further functional studies to clarify their roles in antimicrobial tolerance and resistance. Identifying and characterizing these putative mechanisms and regulons, as well as the possible connections between them, may represent an important step forward leading to novel targets for developing new approaches to tackle carbapenem-resistant pathogens.

## Figures and Tables

**Table 1:** Number of proteins identified with higher or lower abundance according to the established thresholds of fold change and *p*-value.

Comparison	Proteins with Higher Abundance	Protein with Lower Abundance	Total
1/8 MIC vs. NAb	33	28	61
1/4 MIC vs. NAb	48	39	87

MIC (minimal inhibitory concentration) of ertapenem; NAb (no antibiotic).

**Table 2:** Proteins demonstrating higher abundance at maximum antibiotic exposure (1/4 MIC) versus NAb (FC ≥ 1.5 and *p* ≤ 0.05). The abundance levels at 1/8 MIC are also displayed, in order to show the trend in abundance.

		1/8 MIC vs. NAb		1/4 MIC vs. NAb	
Accession No.	Description of the Protein	FC	*p*-Value	FC	*p*-Value
WP_004143727.1	Stationary phase-induced ribosome-associated protein	2.99	0.00	4.63	0.00
WP_004224493.1	phenylacetate-CoA oxygenase subunit PaaI	2.83	0.01	3.31	0.00
WP_004224492.1	1,2-phenylacetyl-CoA epoxidase subunit B	2.49	0.00	2.73	0.00
WP_023302002.1	phenylacetate-CoA oxygenase/reductase subunit PaaK	2.18	0.01	2.50	0.00
WP_002921438.1	C4-dicarboxylate transporter	2.31	0.00	2.49	0.00
WP_002910896.1	YcgN family cysteine cluster protein	1.72	0.06	2.36	0.02
WP_023280306.1	L,D-transpeptidase	1.82	0.00	2.28	0.00
**WP_013263789.1**	**subclass B1 metallo-beta-lactamase VIM-1**	**−1.42**	0.00	**2.25**	0.00
WP_002917960.1	galactarate dehydratase	1.61	0.04	2.19	0.00
WP_004148220.1	1,2-phenylacetyl-CoA epoxidase subunit A	1.84	0.05	2.15	0.00
WP_019705815.1	LysM peptidoglycan-binding domain-containing protein	1.80	0.00	2.08	0.00
WP_004143718.1	hypothetical protein	2.32	0.21	2.04	0.00
WP_000124850.1	50S ribosomal protein L20	1.77	0.06	2.01	0.03
WP_002909082.1	lipoprotein	1.74	0.01	1.93	0.00
WP_110244503.1	carbon starvation-induced protein CsiD	1.81	0.00	1.92	0.00
WP_002907763.1	alkene reductase	1.60	0.03	1.87	0.01
WP_002915259.1	L-serine ammonia-lyase	1.78	0.00	1.85	0.00
WP_023302381.1	2′,3′-cyclic-nucleotide 2′-phosphodiesterase	1.13	0.10	1.84	0.00
WP_023301830.1	ABC transporter substrate-binding protein	1.45	0.00	1.83	0.00
WP_002907918.1	membrane protein	1.88	0.03	1.81	0.02
WP_002915106.1	RNA polymerase sigma factor RpoS	1.87	0.00	1.81	0.00
WP_004185056.1	ethanolamine utilization acetate kinase EutQ	1.86	0.00	1.80	0.00
WP_002916849.1	DUF1190 family protein	1.68	0.02	1.77	0.00
WP_002914189.1	multidrug efflux RND transporter periplasmic adaptor subunit OqxA	1.52	0.00	1.75	0.00
WP_002907759.1	superoxide dismutase SodC2	1.20	0.15	1.74	0.02
WP_002889376.1	ribonuclease HII	1.45	0.03	1.74	0.05
WP_001144069.1	30S ribosomal protein S21	1.36	0.15	1.71	0.05
WP_004151997.1	ethanolamine utilization microcompartment protein EutK	1.45	0.01	1.71	0.00
WP_004152003.1	aldehyde dehydrogenase EutE	1.68	0.00	1.70	0.00
WP_002923306.1	6-phospho-alpha-glucosidase	1.75	0.01	1.70	0.00
WP_004157740.1	iron uptake system protein EfeO	1.41	0.01	1.69	0.00
WP_023301626.1	phosphate acetyltransferase	1.64	0.00	1.69	0.00
WP_004152644.1	single-stranded DNA-binding protein	1.67	0.02	1.68	0.02
WP_002912948.1	hypothetical protein	1.39	0.00	1.68	0.00
**WP_004900870.1**	**tryptophan permease**	**−7.75**	0.00	**1.68**	0.00
WP_023302039.1	peptide ABC transporter substrate-binding protein	1.43	0.00	1.68	0.00
WP_020802835.1	DUF523 domain-containing protein	1.46	0.24	1.66	0.00
WP_002898195.1	L,D-transpeptidase	1.29	0.04	1.66	0.02
WP_023301761.1	U32 family peptidase	1.75	0.02	1.66	0.00
WP_004174759.1	amino acid ABC transporter substrate-binding protein	1.28	0.01	1.65	0.00
WP_002914339.1	multidrug export protein EmrA	1.19	0.02	1.63	0.00
WP_004184243.1	ABC transporter ATP-binding protein	1.49	0.00	1.61	0.01
WP_023301892.1	polyphosphate kinase 2	1.42	0.04	1.59	0.02
WP_004144787.1	nucleoside permease	1.30	0.07	1.53	0.01
WP_004174905.1	ethanolamine utilization microcompartment protein EutL	1.56	0.00	1.53	0.00
WP_002885659.1	RNA-binding protein Hfq	1.44	0.05	1.52	0.00
WP_004174538.1	N-acetylmuramoyl-L-alanine amidase	1.35	0.00	1.52	0.00
WP_002907788.1	cyclopropane fatty acyl phospholipid synthase	1.45	0.00	1.52	0.00

Proteins with opposite trends at different MICs are highlighted in bold. Highlighted in red are proteins that do not pass the threshold of significance at 1/8 MIC. FC (fold change); MIC (minimal inhibitory concentration) of ertapenem; NAb (no antibiotic).

**Table 3:** Proteins demonstrating lower abundance at maximum antibiotic exposure (1/4 MIC) versus NAb (FC ≤ −1.5 and *p* ≤ 0.05). Same proteins are also shown for 1/8 MIC to show the trend in abundance.

Accession No.	Description of the Protein	FC 1/8 MIC /NAb	*p*-Value 1/8 MIC /NAb	FC 1/4 MIC /NAb	*p*-Value 1/4 MIC /NAb
WP_004118241.1	Fe(3+)-dicitrate ABC transporter substrate-binding protein FecB	−2.61	0.02	−2.51	0.01
WP_001287521.1	transcription termination/antitermination protein NusG	−2.40	0.01	−2.46	0.00
WP_004152286.1	LacI family DNA-binding transcriptional regulator	−2.33	0.01	−2.34	0.00
WP_001293886.1	DUF86 domain-containing protein	−2.35	0.00	−2.33	0.00
WP_004152117.1	Hsp20/alpha crystallin family protein	−2.19	0.01	−2.20	0.00
WP_004152102.1	hypothetical protein	−2.27	0,.00	−2.13	0.00
WP_004152116.1	heat shock survival AAA family ATPase ClpK	−2.17	0.00	−2.12	0.00
WP_003159185.1	recombinase family protein	−2.11	0.01	−2.06	0.00
WP_004183942.1	Heat shock protein	−1.56	0.03	−2.04	0.,00
WP_004145074.1	heat shock chaperone IbpB	−1.80	0.04	−2.03	0.00
WP_032488579.1	AAC(6′)-Ib family aminoglycoside 6′-N-acetyltransferase	−2.17	0.07	−1.98	0.00
WP_009483782.1	diguanylate cyclase	−2.09	0.01	−1.98	0.00
WP_003032875.1	Cu(+)/Ag(+) sensor histidine kinase	−2.06	0.00	−1.97	0.,00
WP_110244509.1	choline transporter	−1.93	0.00	−1.95	0.00
WP_000018326.1	aminoglycoside O-phosphotransferase APH(3′)-Ia	−1.97	0.00	−1.94	0.00
WP_004152099.1	arsenical pump-driving ATPase	−1.78	0.00	−1.94	0.01
WP_004098955.1	copper-translocating P-type ATPase	−1.91	0.03	−1.91	0.00
WP_002210514.1	NAD(P)-dependent oxidoreductase	−1.83	0.00	−1.89	0.00
WP_004152280.1	TonB-dependent siderophore receptor	−1.89	0.00	−1.88	0.00
WP_004151523.1	heat shock protein IbpA	−1.70	0.03	−1.88	0.00
WP_004152291.1	ATPase AAA	−1.88	0.00	−1.86	0.00
WP_004152097.1	arsenate reductase	−1.94	0.01	−1.83	0.00
WP_000777555.1	trimethoprim-resistant dihydrofolate reductase DfrA1	−1.90	0.00	−1.82	0.00
WP_004152282.1	iron-dicitrate ABC transporter ATP-binding subunit	−2.13	0.01	−1.82	0.01
WP_004152290.1	ATPase	−1.73	0.02	−1.81	0.01
WP_000523813.1	chromosome-partitioning protein ParA	−1.80	0.00	−1.79	0.00
WP_000259031.1	sulfonamide-resistant dihydropteroate synthase Sul1	−1.71	0.00	−1.78	0.00
WP_001206317.1	ANT(3′′)-Ia family aminoglycoside nucleotidyltransferase AadA1	−1.91	0.00	−1.76	0.00
WP_002907469.1	collagenase-like protease	−1.86	0.00	−1.76	0.00
WP_023302347.1	phosphomethylpyrimidine synthase ThiC	−1.21	0.01	−1.76	0.00
WP_004146300.1	thiamine phosphate synthase	−1.32	0.00	−1.74	0.00
WP_004152079.1	efflux RND transporter periplasmic adaptor subunit	−1.83	0.03	−1.72	0.00
WP_004177871.1	thiazole biosynthesis adenylyltransferase ThiF	−1.21	0.08	−1.64	0.00
WP_004152308.1	thiazole synthase	−1.11	0.11	−1.61	0.00
WP_004152062.1	ParB/RepB/Spo0J family plasmid partition protein	−1.70	0.01	−1.60	0.01
WP_004152103.1	hypothetical protein	−1.70	0.10	−1.56	0.00
WP_001188930.1	DNA-binding response regulator	−1.56	0.04	−1.53	0.01
WP_004152101.1	transcriptional regulator	−1.69	0.11	−1.51	0.04
WP_004152720.1	type II toxin–antitoxin system RelE/ParE family toxin	−1.43	0.05	−1.51	0.00

Highlighted in red are proteins that do not pass the threshold of significance at 1/8 MIC. MIC (minimal inhibitory concentration) of ertapenem; NAb (no antibiotic).

**Table 4:** List of acquired antibiotic resistance genes in *K. pneumoniae* CCUG 70747.

Gene	Name	Conferring Resistance Against (Class-Subclass)
*sul1*	Sulfonamide-resistant dihydropteroate synthase Sul1	Sulfonamide
*aac(6′)-Ib*	AAC(6′)-Ib family aminoglycoside 6′-N-acetyltransferase gene family	Aminoglycosides-Gentamicin
*aph(3′)-Ic*	Aminoglycoside O-phosphotransferase APH(3′′)-Ic	Aminoglycosides-Streptomycin
*dfrA1, ant(3″)-Ia*	Trimethoprim-resistant dihydrofolate reductase DfrA1 gene family	Aminoglycosides-Spectinomycin/Streptomycin
*bla*_VIM-1_	Subclass B1 metallo-beta-lactamase VIM-1	Beta-lactamases-Carbapenem
*bla*_SHV-200_	Class A beta-lactamase SHV-200	Beta-lactamases-Penicililins, cephalosporins
*bla*_KPC-2_	Carbapenem-hydrolyzing class A beta-lactamase KPC-2	Beta-lactamases-Carbapenem
*bla*_TEM-1_	Broad-spectrum class A beta-lactamase TEM-1	Beta-lactamases

## Data Availability

The data sets produced in this study are available in the following databases: Proteomic MS data can be found at the ProteomeXchange with identifier PXD061842.

## Supplementary Materials

The following supporting information can be downloaded at: https://www.mdpi.com/article/10.3390/ijms26188988/s1. Reference [107] is cited in the Supplementary Materials.
