# Supplementary material for: Insights into the Metabolic Adaptations of a Carbapenem-Resistant Klebsiella pneumoniae Strain on Exposure to Sublethal Concentrations of Ertapenem
Source: Int J Mol Sci. 2025 Sep 15;26(18):8988. doi: 10.3390/ijms26188988 (PMC12470040; doi:10.3390/ijms26188988)
Supplement: Supplementary file 1 [file ijms-26-08988-s001.zip › File_S4_Proteomics_protocol.pdf]

File S4. Detailed proteomic experiment for Supporting Information

Proteomic sample preparation

Samples were digested with trypsin using the filter-aided sample preparation (FASP) method (Wiśniewski et al., 2009). Briefly, protein samples were reduced with 100 mM dithiothreitol at 60°C for 30 min, transferred to 30 kDa MWCO Pall Nanosep centrifugal filters (Sigma-Aldrich), washed with 8M urea solution repeatedly and alkylated by addition of methyl methanethiosulfonate to a final concentration of 10 mM. Digestion was performed in 50 mM TEAB, 0.5% sodium deoxycholate (SDC) over night at 37°C after addition of Trypsin (Pierce Trypsin Protease, MS Grade, Thermo Fisher Scientific) in a ratio of 1: 100 relative to protein amount. An additional portion of trypsin were added and incubated for another two hours followed by collection of the peptides by centrifugation. Digested peptides were labelled using TMT 10-plex isobaric mass tagging reagents (Thermo Fisher Scientific) according to the manufacturer instructions. The labelled samples were combined into one pooled sample, concentrated using vacuum centrifugation, and SDC was removed by acidification with 10% trifluoroacetic acid and subsequent centrifugation. The labelled pooled sample was treated with Pierce peptide desalting spin columns (Thermo Fischer Scientific) according to the manufacturer's instructions.

Peptide were fractionated into 20 fraction using reversed-phase XBridge C18 3.5 µm, 3.0x150 mm column (Waters) using 32 min gradient from 3% to 40% solvent B, solvent A was 10 mM ammonium formate buffer at pH 10.0, solvent B was 90% acetonitrile, 10% 10 mM ammonium formate at pH 10.0. The fractions were combined into 10 fractions.

LC-MS3 analysis QMS

The fractions were analysed on an Orbitrap Lumos™ Tribrid™ mass spectrometer interfaced with an Easy-nLC1200 liquid chromatography system (all Thermo Fisher Scientific). Peptides were trapped on an Acclaim Pepmap 100 C18 trap column (100 µm x 2 cm, particle size 5 µm, Thermo Fisher Scientific) and separated on an in-house packed analytical column (35 cm x 75 µm, particle size 3 µm, Reprosil-Pur C18, Dr. Maisch) using a stepped gradient from 4% to 27% acetonitrile in 0.2% formic acid over 77 min at a flow of 300 nl/min, followed by increase to 80% acetonitrile for 13 min. MS scans were performed at 120 000 resolution and an m/z range of 375-1375. MS/MS analysis was performed in data-dependent mode, using a top speed cycle of 3 s for the most intense precursor ions with a charge state of 2-7. Precursor ions were isolated in the quadrupole with a 0.7 m/z isolation window and dynamic exclusion of fragmented precursors was set to 10 ppm for a duration of 45 s. Isolated precursor ions were subjected to collision induced dissociation (CID) with collision energy set to 35

and a maximum injection time of 50 ms. The MS2 fragment ions were detected in the ion trap followed by multi-notch (simultaneous) isolation of the top 10 abundant fragment ions for further fragmentation (MS3) by higher-energy collision dissociation (HCD) at 65% and detection in the Orbitrap at 50 000 resolutions, m/z range of 100-500.

Proteomic data analysis

Raw files were processed and analysed with Proteome Discoverer (V. 2.4, Thermo Scientific). The data was matched against a database containing the proteome from the strain included in the study (*K. pneumoniae* CCUG 70747 database, Supplementary information) using Mascot 2.5 (Matrix Science) as a search engine with a precursor tolerance of 5 ppm and a fragment ion tolerance of 0.05 Da. Tryptic peptides were accepted with zero missed cleavage. Methionine oxidation was set as a variable modifications and cysteine alkylation, TMT on lysine and peptide N-termini were set as fixed modifications. Percolator was used for the validation of identified proteins. TMT reporter ions were identified in the MS3 HCD spectra with 3 mmu mass tolerance, and the TMT reporter intensity values for each sample were normalized on the total peptide amount. The quantified proteins were filtered at 1% FDR and grouped by sharing the same sequences to minimize redundancy. Unique peptides for a given protein were considered for quantification of the proteins Percolator was used for PSM validation at an FDR of 1% and the identified proteins were filtered at 1% FDR.

Acknowledgment

Proteomic analysis was performed at the Proteomics Core Facility, Sahlgrenska academy, Gothenburg University.

Reference

Wiśniewski, J.R.; Zougman, A.; Nagaraj, N.; Mann, M. Universal sample preparation method for proteome analysis. *Nat Methods*. **2009**;6(5):359-362. doi:10.1038/nmeth.1322
