# Supplementary material for: Insights into the Metabolic Adaptations of a Carbapenem-Resistant Klebsiella pneumoniae Strain on Exposure to Sublethal Concentrations of Ertapenem
Source: Int J Mol Sci. 2025 Sep 15;26(18):8988. doi: 10.3390/ijms26188988 (PMC12470040; doi:10.3390/ijms26188988)
Supplement: Supplementary file 1 [file ijms-26-08988-s001.zip › Supplementary_tables.pdf]

Table S1- Minimum inhibitory concentration (MIC) profile of K- pneumoniae CCUG 70747.

**Antibiotic**	**MIC (mg/L)**
Amikacin	16
Amoxicillin-clavulanic acid (fixed 2 mg/L clavulanic acid)	>128
Amoxicillin	>32
Ampicillin	>32
Cefepime	>16
Cefotaxime	>8
Ceftazidime	>16
Ceftibuten	>4
Ceftriaxone	>4
Cefuroxime	>16
Cephalexin	>32
Ciprofloxacin	>4
Colistin	≤0.25
Ertapenem	>4
Gentamicin	1
Imipenem	16
Levofloxacin	>4
Meropenem	>16
Nitrofurantoin	64
Piperacillin-tazobactam (fixed 4 mg/L tazobactam)	>64
Temocillin	>128
Tigecycline	0.25
Tobramycin	16
Trimethoprim	>16
Trimethoprim-sulfamethoxazole	>16

Table S2. Minimum inhibitory concentration (MIC) determination for K. pneumoniae CCUG 70747 and ertapenem.

	Concentration of ertapenem (µg/ml)											
	1000	500	250	125	62.5	31.25	15.625	7.81	3.91	1.95	0	C-
Col	0.00125	-0.00275	0.00125	-0.00275	-0.00075	0.30625	0.31825	0.35525	0.36325	0.35825	0.37225	-0.00475
Col	0.00025	-0.00575	-0.00075	-0.00175	-0.00175	0.26725	0.30725	0.34425	0.38725	0.34225	0.40525	0.00025
Mix	0.01025	-0.00075	0.00025	0.00325	-0.00075	0.28325	0.30025	0.35625	0.33825	0.36125	0.40025	0.00225
Mix	0.00725	0.00025	0.00425	0.00125	0.00125	0.26625	0.28425	0.32225	0.33925	0.37725	0.45325	0.00225
	-0.00075	0.00225	0.00225	0.00225	0.00225	0.00525	0.00325	0.00425	0.00425	0.00725	0.00725	0.00525
	0.00025	0.00425	0.00425	0.00325	0.00225	0.00425	0.00225	0.00525	0.00325	0.00825	0.00625	0.00625
	0.00225	0.00425	0.00225	0.00425	0.00225	0.00525	0.00725	0.00525	0.00525	0.00525	0.00225	0.00425
	0.00225	0.00525	0.00425	0.00425	0.00225	0.00725	0.00625	0.00525	0.00225	0.00725	0.00525	0.00525

Table S3. COG category nomenclature and number of proteins over- or underexpressed in each category.

# Code	Functional category	Under	Over
J	Translation, ribosomal structure and biogenesis	1	2
A	RNA processing and modification		
K	Transcription	5	1
L	Replication, recombination and repair	1	3
B	Chromatin structure and dynamics		
D	Cell cycle control, cell division, chromosome partitioning	1	0
Y	Nuclear structure		
V	Defense mechanisms	0	1
T	Signal transduction mechanisms	2	1
M	Cell wall/membrane/envelope biogenesis	1	6
N	Cell motility		
Z	Cytoskeleton		
W	Extracellular structures		
U	Intracellular trafficking, secretion, and vesicular transport	1	1
O	Posttranslational modification, protein turnover, chaperones	6	1
X	Mobilome: prophages, transposons		
C	Energy production and conversion	0	6
G	Carbohydrate transport and metabolism	0	2
E	Amino acid transport and metabolism	0	6
F	Nucleotide transport and metabolism		
H	Coenzyme transport and metabolism	4	1
I	Lipid transport and metabolism	1	0
P	Inorganic ion transport and metabolism	8	3
Q	Secondary metabolites biosynthesis, transport and catabolism	0	2
R	General function prediction only		
S	Function unknown	4	6

Table S4. Regulatory proteins identified by PR2P that show significant variation in their expression.

**TCS**	**Two component system**							
**Gene**	**Description**	**FC**	**P-value**	**class**	**type**	**GO IDs**	**GO names**	**P2RP description**
WP_001188930.1	MULTISPECIES: DNA-binding response regulator [Gammaproteobacteria]	-1.53	0.01	RR	OmpR	P:GO:0000160; P:GO:0006355; F:GO:0003677; C:GO:0005737	P:phosphorelay signal transduction system; P:regulation of transcription, DNA-templated; F:DNA binding; C:cytoplasm	Response regulator, OmpR family contains 1 Response_reg,1 Trans_reg_C
WP_003032875.1	MULTISPECIES: Cu(+)/Ag(+) sensor histidine kinase [Enterobacterales]	-1.97	0.00	HK	Classic	P:GO:0000160; P:GO:0018106; F:GO:0000155; F:GO:0005524; C:GO:0005886; C:GO:0016021	P:phosphorelay signal transduction system; P:peptidyl-histidine phosphorylation; F:phosphorelay sensor kinase activity; F:ATP binding; C:plasma membrane; C:integral component of membrane	Histidine kinase, Classic contains 1 HAMP,1 HisKA,1 HATPase_c
**TF**	**Transcription factors**							
**Gene**	**Description**	**FC**	**P-value**	**class**	**type**	**GO IDs**	**GO names**	**P2RP description**
WP_002915106.1	**MULTISPECIES: RNA polymerase sigma factor RpoS [Enterobacterales]**	1.81	0.00	SF		P:GO:0043254; P:GO:2000112; P:GO:2000142; F:GO:0001000; F:GO:0003677; F:GO:0016987; C:GO:0005737	P:regulation of protein-containing complex assembly; P:regulation of cellular macromolecule biosynthetic process; P:regulation of DNA-templated transcription, initiation; F:bacterial-type RNA polymerase core enzyme binding; F:DNA binding; F:sigma factor activity; C:cytoplasm	**Sigma factor, RpoE family contains 1 Sigma70_r1_2,1 Sigma70_r2,1 Sigma70_r3,1 Sigma70_r4**
WP_004151997.1	MULTISPECIES: ethanolamine utilization microcompartment protein EutK [Enterobacterales]	1.71	0.00	TR	FaeA	P:GO:0010165; C:GO:0031469	P:response to X-ray; C:bacterial microcompartment	Transcription factor, FaeA family contains 1 FaeA
WP_004152101.1	MULTISPECIES: transcriptional regulator [Enterobacterales]	-1.51	0.04	TR	ArsR	P:GO:0006355; P:GO:0046685; P:GO:2000112; F:GO:0003677; F:GO:0003700	P:regulation of transcription, DNA-templated; P:response to arsenic-containing substance; P:regulation of cellular macromolecule biosynthetic process; F:DNA binding; F:DNA-binding transcription factor activity	Transcription factor, ArsR family contains 1 HTH_5
WP_001188930.1	MULTISPECIES: DNA-binding response regulator [Gammaproteobacteria]	-1.53	0.01	RR	OmpR	P:GO:0000160; P:GO:0006355; F:GO:0003677; C:GO:0005737	P:phosphorelay signal transduction system; P:regulation of transcription, DNA-templated; F:DNA binding; C:cytoplasm	Response regulator, OmpR family contains 1 Response_reg,1 Trans_reg_C

WP_016947274.1	MULTISPECIES: transcriptional regulator PtsJ [Enterobacteriaceae]	-1.58	0.10	OCS	GntR	P:GO:0006355; F:GO:0003700; F:GO:0003824; F:GO:0030170	P:regulation of transcription, DNA-templated; F:DNA-binding transcription factor activity; F:catalytic activity; F:pyridoxal phosphate binding	Transcription factor, GntR family contains 1 GntR,1 Aminotran_1_2
WP_000523813.1	MULTISPECIES: chromosome partitioning protein ParA [Enterobacteriaceae]	-1.79	0.00	TR	MerR	0	0	Transcription factor, MerR family contains 1 MerR
WP_004118235.1	MULTISPECIES: RNA polymerase sigma factor [Enterobacterales]	-2.17	0.00	SF	FecI	P:GO:0006950; P:GO:2000142; F:GO:0003677; F:GO:0016987	P:response to stress; P:regulation of DNA-templated transcription, initiation; F:DNA binding; F:sigma factor activity	Sigma factor, Ecf family contains 1 Sigma70_r2,1 Sigma70_r4
WP_004152286.1	MULTISPECIES: LacI family DNA-binding transcriptional regulator [Enterobacterales]	-2.34	0.00	OCS	LacI	P:GO:0010629; P:GO:0045892; P:GO:2000113; F:GO:0000987; F:GO:0001217; F:GO:0042802	P:negative regulation of gene expression; P:negative regulation of transcription, DNA-templated; P:negative regulation of cellular macromolecule biosynthetic process; F:cis-regulatory region sequence-specific DNA binding; F:DNA-binding transcription repressor activity; F:identical protein binding	Transcription factor, LacI family contains 1 LacI,1 Peripla_BP_1
