# Supplementary figures and images for: Insights into the Metabolic Adaptations of a Carbapenem-Resistant Klebsiella pneumoniae Strain on Exposure to Sublethal Concentrations of Ertapenem
Source: Int J Mol Sci. 2025 Sep 15;26(18):8988. doi: 10.3390/ijms26188988 (PMC12470040; doi:10.3390/ijms26188988)

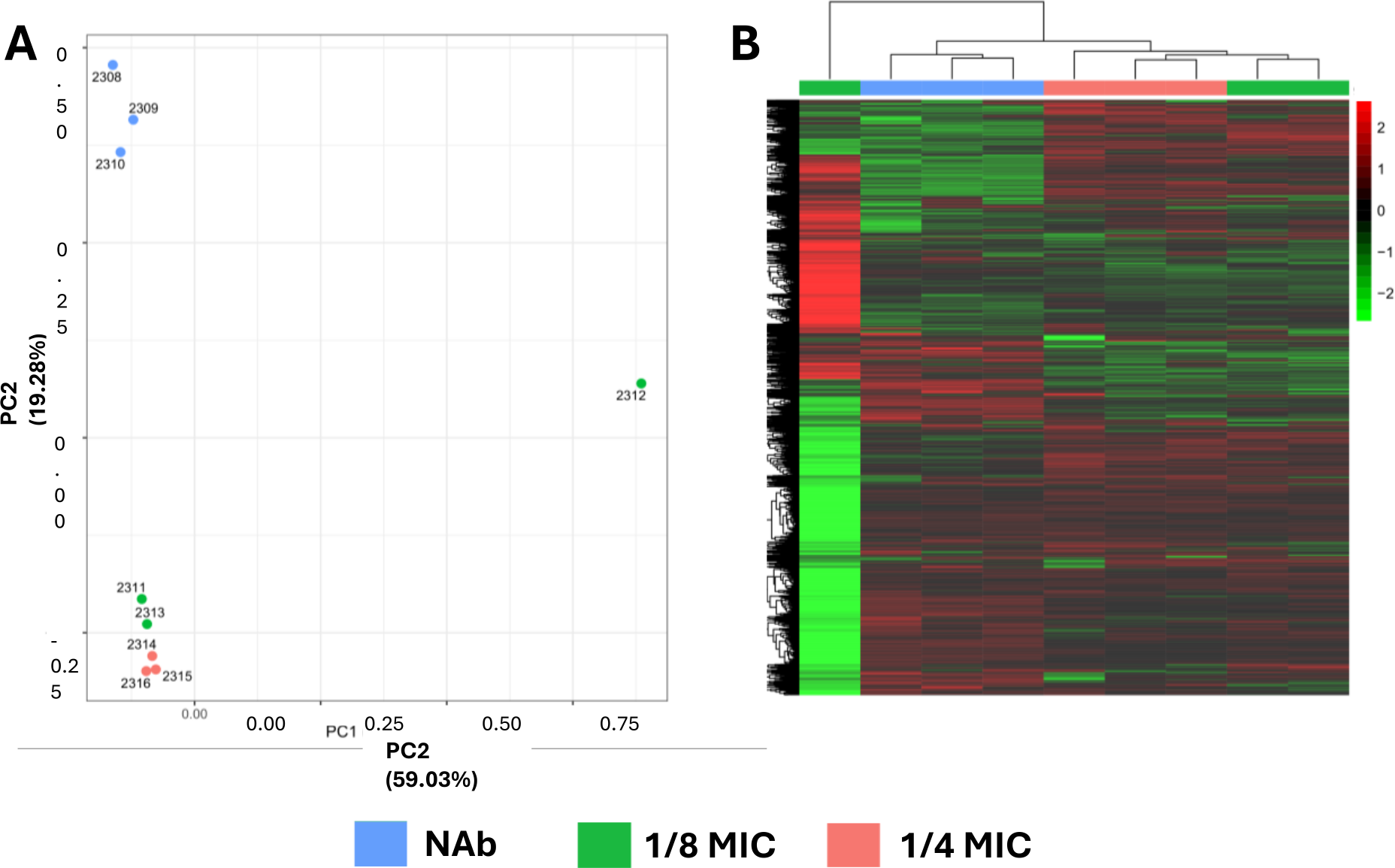

Supplement: Supplementary file 1 [file ijms-26-08988-s001.zip › Figure_S1.tiff]
